# From human business to machine learning—methods for automating real estate appraisals and their practical implications

**DOI:** 10.1365/s41056-022-00063-1

**Published:** 2022-07-19

**Authors:** Moritz Stang, Bastian Krämer, Cathrine Nagl, Wolfgang Schäfers

**Affiliations:** International Real Estate Business School, University of Regensburg, Regensburg, Germany

**Keywords:** Automated Valuation Models, Extreme Gradient Boosting, Housing Market, Machine Learning, Sales Comparison Method, Automated Valuation Models, Extreme Gradient Boosting, Wohnungsmarkt, Machine Learning, Vergleichswertverfahren

## Abstract

Until recently, in most countries, the use of Automated Valuation Models (AVMs) in the lending process was only allowed for support purposes, and not as the sole value-determining tool. However, this is currently changing, and regulators around the world are actively discussing the approval of AVMs. But the discussion is generally limited to AVMs that are based on already established methods such as an automation of the traditional sales comparison approach or linear regressions. Modern machine learning approaches are almost completely excluded from the debate. Accordingly, this study contributes to the discussion on why AVMs based on machine learning approaches should also be considered. For this purpose, an automation of the sales comparison method by using filters and similarity functions, two hedonic price functions, namely an OLS model and a GAM model, as well as a XGBoost machine learning approach, are applied to a dataset of 1.2 million residential properties across Germany. We find that the machine learning method XGBoost offers the overall best performance regarding the accuracy of estimations. Practical application shows that optimization of the established methods—OLS and GAM—is time-consuming and labor-intensive, and has significant disadvantages when being implemented on a national scale. In addition, our results show that different types of methods perform best in different regions and, thus, regulators should not only focus on one single method, but consider a multitude of them.

## Abstract (de)

Bis vor Kurzem war der Einsatz von automatisierten Bewertungsmodellen (AVMs) im Rahmen der kreditwirtschaftlichen Immobilienbewertung in den meisten Ländern lediglich zu Unterstützungszwecken erlaubt. Ein alleiniger Einsatz dieser zur Wertfindung blieb in der Regel untersagt. Dies ändert sich jedoch aktuell und Regulierungsbehörden weltweit setzen sich mit deren Zulassung auseinander. Die Diskussion beschränkt sich dabei jedoch oftmals auf bereits etablierte Methoden zur Automatisierung. Diese umfassen beispielsweise eine automatisierte Version des traditionellen Vergleichswertverfahrens oder lineare Regressionsmodelle. Moderne Ansätze aus dem Bereich des maschinellen Lernens werden in der Diskussion fast gänzlich außer Acht gelassen. Diese Studie liefert einen relevanten Beitrag zur Diskussion, warum künftig auch AVMs, die auf Ansätzen des maschinellen Lernens basieren, im Rahmen der Debatte berücksichtigt werden sollten. Zu diesem Zweck werden eine Automatisierung des traditionellen Vergleichswertverfahrens unter Verwendung von Filtern und Ähnlichkeitsfunktionen, zwei hedonische Preisfunktionen – ein OLS- und ein GAM-Modell – sowie ein XGBoost-Ansatz des maschinellen Lernens auf einen Datensatz von 1,2 Mio. Wohnimmobilien in Deutschland angewendet. Unsere Analyse zeigt, dass die maschinelle Lernmethode XGBoost insgesamt die höchste Genauigkeit hinsichtlich der Marktwertschätzung aufweist. Die Umsetzung zeigt darüber hinaus, dass die Optimierung der etablierten Methoden – OLS und GAM – zeit- und arbeitsintensiv ist und erhebliche Nachteile bei einer Implementierung auf nationaler Ebene mit sich bringt. Darüber hinaus zeigen unsere Ergebnisse, dass verschiedene Arten von Methoden in verschiedenen Regionen am besten Abschneiden und Regulierungsbehörden sich daher nicht nur auf eine einzige Methode konzentrieren, sondern immer mehrere in Betracht ziehen sollten.

## Introduction

Although the real estate industry is often accused of lagging behind in terms of digitalization, the automation of processes is in fact being more and more actively discussed. In addition to the potential cost savings, ongoing improvements of computer resources and available data play an important role. Hence, it is now possible to raise data potential by automating daily processes. This potential can be leveraged in all areas of the real estate industry. Focusing on valuation, Automated Valuation Models (AVMs) have the power to change the appraisal process in many ways.

In the real estate industry, there are three different approaches to assessing properties, namely the cost approach, the income approach, and the sales comparison approach (see, e.g., Schulz et al. (2014) and Kim et al. (2020)). The latter assumes that the value of a property can be derived from the value of comparable properties, and is particularly well suited for automated real estate valuations. Various ways are known in science and practice to apply the sales comparison approach in the context of AVMs (see, Isakson (2002)). Besides the integration of filters and similarity functions, well-established hedonic price models and modern machine learning approaches can also be used for AVM construction (see, e.g., Pagourtzi et al. (2003) and Bogin and Shui (2020)). Furthermore, repeated sales methods are employed for AVM applications, see, e.g., Oust et al. (2020).

Currently, the use of AVMs in the lending process is only allowed for supporting purposes in most countries and not as a value-determining tool (Matysiak (2017) and Downie and Robson (2008)). Although there are now regulatory efforts to include AVMs in the lending process, this is only possible if the traceability, auditability, robustness and resilience of the inputs and outputs can be guaranteed (European Banking Authority (2020)). However, it remains unclear which of the abovementioned methods meet these requirements. While there is an ongoing debate about allowing the use of AVMs based on already established methods such as similarity functions or OLS regressions within the lending process, the application of modern machine learning methods is almost completely absent from the regulatory discussion. This is in fact due to the “black box” label of modern machine learning techniques. The decisions made by these methods are not as easy to understand as is the case for linear-based models due to more complex internal processes. However, in recent years, there have been various approaches to opening this black box; see for example by Friedman (2001), Goldstein et al. (2015), Lundberg and Lee (2017) and Apley and Zhu (2020). Through these approaches, the requirements of the supervisory authority for tractability and audibility can be considered.

Therefore, the question arises as to whether modern machine learning algorithms should also be considered by the regulatory body. The objective of this paper is to contribute to this ongoing debate and deliver further insights, based on a unique nationwide dataset, into the optimal use of modern machine learning algorithms for AVMs from a theoretical and practical point of view. For this purpose, an automation of the sales comparison method by using filters and similarity functions, referred to as Expert Function (EXF), two hedonic price functions based on Ordinary Least Squares (OLS) and Generalized Additive Models (GAM), as well as the machine learning approach eXtreme Gradient Boost (XGBoost), are compared with each other.

We are the first to use a unique dataset of around 1.2 million market values of standard residential properties across Germany between 2014 and 2020, provided by a large German Banking Group, to test the four selected AVM approaches with respect to the question of whether the application of modern machine learning algorithms on a nationwide level is superior to the other approaches. The market values are based on appraiser valuations and can thus be assumed to be objective property values—unlike, for example, listing data.

The German real estate market is characterized by many different local markets whose development is often mutually independent. While metropolitan regions have seen a significant rise in values in recent years, property values in rural areas have stagnated in some cases. We are therefore also interested in whether there is one type of model which performs best in varying submarkets or whether there are structural differences. Due to the low population density, fewer observations are available in rural areas, which also raises the question of whether data availability has an impact on model performance and whether this has an influence on the decision to use machine learning algorithms for AVMs or not.

Hence, we contribute to the literature by addressing the following three research questions:

I. Do machine learning methods outperform well-established AVM methods like the OLS, the GAM and the EXF, and should they therefore also be considered within the regulatory discussion of AVMs?
II. Should AVMs rely on the use of one single approach, or should multiple models be integrated for different spatial areas?
III. Does the performance of the methods depend on data availability and structure?

Although AVMs represent a wide field in the literature, we are—to the best of our knowledge—the first to compare a filter- and similarity-based AVM approach, two well-established hedonic methods and a modern machine learning approach on a nation-wide level. Our results provide important insights into the practical application of AVMs and the discussion as to whether the usage of machine learning algorithms for the lending process should be allowed from a regulatory perspective.

We find that the machine learning method XGBoost offers the best performance regarding estimation accuracy. The EXF provides the highest transparency, but lower accuracy, as it tends to underestimate and does not allow calculation of the influences of individual property characteristics. The OLS and GAM are capable of doing so, but are most often outperformed by the XGBoost. Another advantage of the XGBoost is its high flexibility. While the optimization of the OLS and the GAM must be mainly done manually to achieve good model performance, the XGBoost automatically detects relevant patterns in the data. Therefore, this algorithm is better suited in practice to performing estimations based on large and complex datasets, such as nation-wide real estate valuations. However, our results also show that it is not advisable to focus on only one method when designing a nation-wide AVM. Although the XGBoost performs best across Germany, there are also regions where the EXF, the OLS or the GAM perform best. In this respect, the data availability within regions plays an important role and it is apparent that the strength of the machine learning approach cannot be improved in regions with limited training data. We therefore generally recommend testing several algorithms per region before making a final choice. In summary, our study shows that the use of machine learning algorithms for AVMs is beneficial in many situations and therefore, their approval should indeed be discussed by the regulatory authorities.

## Literature review

The following section provides a general overview of the existing literature in the field of AVMs. Due to the generally high attention devoted to this topic by the scientific community, numerous publications can be found dealing with AVMs.

The sales comparison approach normally uses a limited set of similar properties to evaluate the market value of a property, as described by French and Gabrielli (2018). Since the beginning of the computer assisted mass appraisal (CAMA) era, this approach has been automated by various researchers and is widely used in practice, especially in North America and the UK. Usually, the designed approaches follow a predefined process to identify the *n* most comparable sales properties from a set of *N* observations. The final estimation is then calculated by taking the mean or similarity-weighted mean of these comparable sales prices. Early adoptions of the similarity-based finding of comparable properties can be found in Underwood and Moesch (1982), Thompson and Gordon (1987), Cannaday (1989), McCluskey and Anand (1999) and Todora and Whiterell (2002). More recently, Brunauer et al. (2017) design an approach for valuations of self-used property based on the sales comparison method. Trawinski et al. (2017) examine the accuracy of two expert algorithms, using either the N‑Latest Transactions in an area (LTA) or the N‑Nearest Similar Properties (NSP), and compare their results with different data-driven regression models. Ciuna et al. (2017) create an approach to overcome the limitations of AVMs in markets with less available data, by means of measuring the similarity degree of the comparables. Kim et al. (2020) automate the sales comparison method to evaluate apartments in Korea and find that their approach outperforms machine learning methods. Larraz et al. (2021) use a computer-assisted expert algorithm and consider differences in characteristics compared to similar properties and their relative location.

As Borst and McCluskey (2007) show, the similarity-based automation of the sales comparison approach is also reflected in spatial autoregressive (SAR) models. The authors state that the automated sales comparison approach can be seen as a special case of a spatially lagged weight matrix model, and that there is also a less formal but clear relationship with geographically weighted regressions (GWR). Applications of SAR models can be found, among other, in McCluskey et al. (2013) and Schulz and Wersing (2021). Compared to the approach of similarity-based finding of comparable properties, the SAR model is a much more complex approach and is associated with a higher computing cost.

The hedonic price function is a well-established model that has been widely used in research for decades and was primary described by Rosen (1974). Hedonic price models do not start from the property to be valued, but from the existing information on any property available in the market, as outlined by Maier and Herath (2015). Accordingly, the property value comprises an aggregation of various attributes or characteristics regarding the amenities, micro/macro location and geodata. This also allows conclusions to be drawn about the influence of individual attributes on the value. Based on Ordinary Least Square Regression (OLS), various studies use this method in real estate valuation, for example Malpezzi (2003), Sirmans et al. (2005) and Schulz et al. (2014). In the most recent studies, OLS is used as a benchmark, for example by Zurada et al. (2011), Chrostek and Kopczewska (2013), Cajias et al. (2019) and Chin et al. (2020). For the interested reader, Metzner and Kindt (2018) and Mayer et al. (2019) provide a detailed literature review of OLS in real estate valuation.

One main disadvantage of the OLS is the dependence on the correctly specified form of the independent variables, as described by Mason and Quigley (1996). As an advanced regression model, the GAM can overcome this drawback, as it can model non-linear relationships. So-called splines are used to non-parametrically describe the relationship between the dependent and independent variables. The model was first introduced by Hastie and Tibshirani (1990) and is based on the Generalized Linear Model established by Nelder and Wedderburn (1972). Investigating the housing market in Los Angeles, Mason and Quigley (1996) are the first to use a GAM in a real estate context and find statistically significant advantages compared to OLS models. The greater flexibility and increased accuracy enable GAMs to gain further acceptance in real estate price estimation. Various other studies deal with the application of GAMs for real estate valuation, namely Pace (1998), Bao and Wan (2004), Bourassa et al. (2007), Bourassa et al. (2010) and Brunauer et al. (2010). For a detailed literature review, see Cajias and Ertl (2018).

Improved data availability and computational power have led to a whole new wave of machine learning methods, and their application to AVMs has become a widely discussed topic within academia. Machine learning methods are designed to identify non-linear structures. In addition to Artificial Neural Networks (ANN) and Support Vector Machines (SVM), tree-based models are most applied in the context of AVMs.

The idea of tree-based models dates back to Morgan and Sonquist (1963) and their automatic interaction detection (AID). The first decision tree algorithm was introduced by Quinlan (1979). The currently most commonly cited and used algorithm for decision trees was introduced by Breiman et al. (1984). Single decision trees are associated with the disadvantage that they easily overfit and therefore might perform worse on unseen data. To overcome this problem, ensemble learning techniques are used (Prajwala (2015)). Ensemble learning is defined as the combination of many “weak-learners” (e.g., single regression trees) to form one single “strong learner” (Sagi and Rokach (2018)). One efficient and commonly used version is the gradient boosting technique. The idea of gradient boosting originates back to Breiman (1997) and was primary introduced for regression trees by Friedman (2001). As Kok et al. (2017) describe, gradient-boosting models build many small decision trees subsequently, from residual-like measures of the previous trees and each tree is built from a random subsample of the dataset. Applied in real estate context, Ho et al. (2021) evaluate property prices in Hong Kong using gradient boosting trees and find that this approach outperforms other machine learning techniques like Support Vector Machines (SVM). Another example can be derived from Singh et al. (2020). The authors compare the result of gradient boosting machines with the results of a random forest regression and a linear regression approach for housing sale data in Ames, Iowa. Their findings confirm the superiority of the gradient boosting approach. Other examples can be found at Pace and Hayunga (2020) and Tchuente and Nyawa (2021). Based on the concept of gradient boosting, Tianqi and Guestrin (2016) implement the eXtreme Gradient Boosting (XGBoost) algorithm. The XGBoost is a computationally effective and highly efficient version of gradient boosting trees and applies a more regularized model structure, in order to control overfitting. Since its introduction it has often been used to tackle real-estate-specific problems. Kumkar et al. (2018), for example, compare four tree-based ensemble methods, namely bagging, random forest, gradient boosting and eXtreme gradient boosting, in terms of their efficiency in the appraisal of property in Mumbai, India. Their findings show that the XGBoost model performs better than to the other models. Sangani et al. (2017) compare the results of different gradient boosting specifications with a simple linear regression. Their analysis is based on a dataset of 2,985,217 parcels in three different counties of California. The XGBoost gradient boosting specification significantly outperforms the linear regression and is also able to perform better than almost all other specifications. Further applications of the XGBoost algorithm can be seen in Kok et al. (2017), Cajias et al. (2019) and Birkeland et al. (2021).

Although AVMs represents a wide field in the literature—to the best of our knowledge—there is currently no research comparing the performance of an advanced machine learning approach with both a filter- and similarity-based AVM and a well-established hedonic model on a nation-wide level. To address this gap in the literature, we design our own filter- and similarity-based AVM, named EXF, and apply two frequently used hedonic models, to compare their results against the performance of a modern machine learning algorithm. We use the XGBoost as our machine learning model. In several other studies, the XGBoost shows encouraging results and, compared to ANNs and SVMs, has the advantage that calculation is quicker and is therefore best suited for the size of our data set. For the hedonic models, we decide to use an OLS and a GAM. The OLS is considered to be the most widely used method in the field of AVMs and is commonly used as a benchmark. Therefore, its results are easy for readers to understand, interpret and classify. The GAM is a further development of the OLS, which can consider non-linearities by means of splines. The results of the GAM are therefore an important extension to those of the OLS. The GAM also demonstrates good performance in many other studies. Our comparison allows us to provide important insights with respect to the practical application of AVMs and the discussion on whether the usage of machine learning algorithms for the lending process should be allowed from a regulatory perspective or not.

## Data

Our analysis is based on a data set of 1,212,546 residential properties across Germany. The data set is provided by a large German banking group and originates from valuations of standard residential real estate lending. The data was collected between 2014 and 2020. Table 1 shows how the observations are distributed over time. As the numbers show, there is a slight decreasing trend which is caused by market fluctuations. Especially, in 2020 due to COVID-19 restrictions, fewer valuations took place.

**Table 1:** Observations per year

	2014	2015	2016	2017	2018	2019	2020
*n*	196,318	196,403	176,238	163,365	165,106	165,996	149,120
*(%)*	0.1619	0.1620	0.1453	0.1347	0.1362	0.1369	0.1230

All properties are georeferenced, making it possible to add a spatial gravity layer in order to account for spatial information. Features describing the location and neighborhood of the observations are added via Open Street Map and Acxiom^1^. The dataset was cleaned for possible outliers, erroneous values, and incompleteness.

**Fig. 1:**
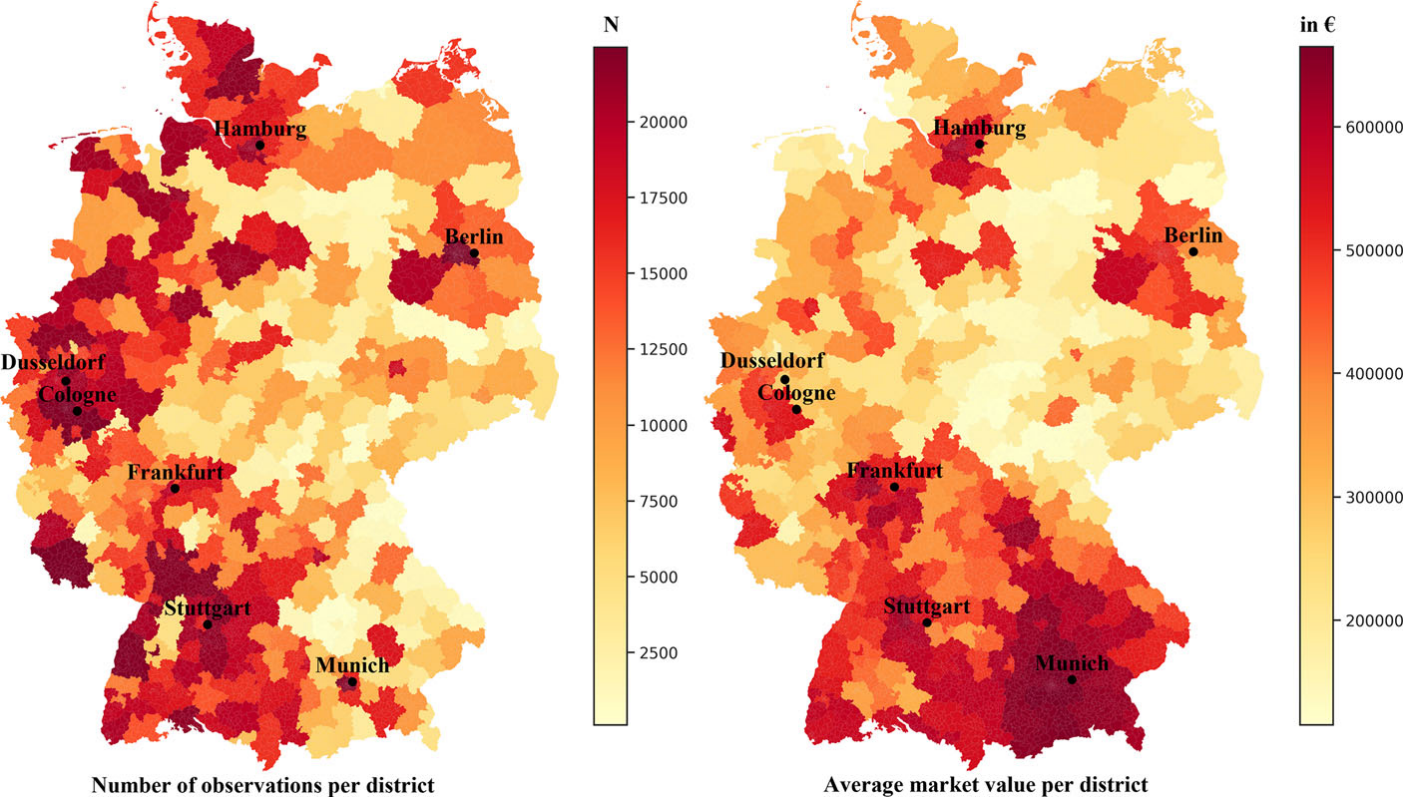
Number of observations and average market value per district

The observations are distributed across Germany and categorized into 327 administrative districts. The division of these regions is aligned with the NUTS‑3 nomenclature of the European Union. The exact distribution of individual observations can be seen on the left side of Fig. 1. Most observations are located around the largest German metropolitan areas like Berlin, Hamburg and Munich. In addition, a difference can be observed between west and east Germany, with the east tending to have fewer observations. This is consistent with the widely diverging population figures between these regions. A comprehensive introduction to the structure of the German regions can be found at Just and Schaefer (2017), and a more detailed overview of the German real estate markets is given by Just and Maennig (2012).

The market value of the properties, based on professional appraiser valuations, is used as the target variable. In contrast to listing data, market values do not depend on subjective seller perceptions of value, but are assessed objectively by outside third parties. An overview of the average market values across the 327 administrative districts is provided on the right side of Fig. 1. The areas with the highest market values can be found in the so-called Top‑7^2^ cities and their commuter belts. Furthermore, the market values are by far the highest in the south of Germany and tend to be lower in the east.

Table 2 shows the features included in our models and summarizes their univariate distributions. In principle, features describing the structural characteristics, micro-location and macro-location of the properties are selected. In addition, the year and quarter of the valuation is used to capture a temporal trend and seasonality. There are no correlations of concern within the data set, so that all variables can be integrated accordingly.^3^

**Table 2:** Descriptive statistics

Variable	Unit	Mean	Median	Standard Deviation	Maximum	Minimum
Market value	Integer	228,157.10	200,000.00	141,717.54	3,860,000.00	20,100.00
Modernization year	Integer	1989.10	1988.00	17.19	2020.00	1950.00
Construction year	Integer	1978.48	1981.00	29.77	2020.00	1900.00
Year of valuation	Integer	2016.82	2017.00	2.00	2020.00	2014.00
Quarter of valuation	Integer	2.45	2.00	1.12	4.00	1.00
Quality grade	Integer	3.12	3.00	0.51	5.00	1.00
Macro score	Float	47.61	47.03	11.20	86.50	9.77
Micro score	Float	72.73	74.20	14.44	99.85	0.00
Living area	Float	120.31	114.68	51.69	440.00	15.00
Lot size	Float	436.48	323.00	541.66	10,000.00	0.00
Latitude	Float	50.62	50.74	1.85	55.02	47.40
Longitude	Float	9.25	8.94	1.90	19.25	5.87
Basement condominium	Binary	0.38	0.00	0.48	1.00	0.00
No basement	Binary	0.19	0.00	0.39	1.00	0.00
Basement	Binary	0.44	0.00	0.50	1.00	0.00
Owner-occupied & Non-owner-occupied	Binary	0.09	0.00	0.29	1.00	0.00
Owner-occupied	Binary	0.70	1.00	0.46	1.00	0.00
Non-owner-occupied	Binary	0.21	0.00	0.41	1.00	0.00
Object subtype condominium	Binary	0.38	0.00	0.48	1.00	0.00
Object subtype detached house	Binary	0.42	0.00	0.49	1.00	0.00
Object subtype not a detached house	Binary	0.20	0.00	0.40	1.00	0.00
Condition good	Binary	0.38	0.00	0.49	1.00	0.00
Condition disastrous	Binary	0.00	0.00	0.02	1.00	0.00
Condition middle	Binary	0.45	0.00	0.50	1.00	0.00
Condition moderate	Binary	0.02	0.00	0.14	1.00	0.00
Condition bad	Binary	0.00	0.00	0.05	1.00	0.00
Condition very good	Binary	0.15	0.00	0.36	1.00	0.00
Regiotype agglo commuter belt	Binary	0.15	0.00	0.36	1.00	0.00
Regiotype agglo CBD	Binary	0.13	0.00	0.34	1.00	0.00
Regiotype agglo middle order centre	Binary	0.13	0.00	0.34	1.00	0.00
Regiotype agglo upper order centre	Binary	0.04	0.00	0.19	1.00	0.00
Regiotype rural commuter belt	Binary	0.15	0.00	0.36	1.00	0.00
Regiotype rural middle order centre	Binary	0.07	0.00	0.26	1.00	0.00
Regiotype rural upper order centre	Binary	0.01	0.00	0.07	1.00	0.00
Regiotype urban commuter belt	Binary	0.15	0.00	0.36	1.00	0.00
Regiotype urban middle order centre	Binary	0.10	0.00	0.29	1.00	0.00
Regiotype urban upper order centre	Binary	0.07	0.00	0.26	1.00	0.00

*Note:* The parameter “market value” is the dependent variable in the model estimation

Features describing the structural characteristics of the properties include the subtype of property, year of construction, modernization year, living area, lot size (only used if the property was not a condominium), use of the property, quality grade, condition and a variable denoting whether the property has a basement or not.

The subtype of a property can be either a “Condominium”, “Detached house” or “Not a detached house”. The year of modernization represents the year in which the last major refurbishment took place. The use of the building describes the possible uses, either “Owner-occupied & Non-owner-occupied”^4^, “Owner-Occupied” or “Non-owner-occupied”. Basically, the variable describes whether a property can be rented to a third-party or not. The quality of the property is measured via a grade, on a scale ranging from 1 (very poor) to 5 (very good). The general condition of the property is represented by a categorial variable with 5 different categories ranging from disastrous to very good. The variable “Basement condominium” measures whether an apartment has an extra cellar compartment or not, whereas the “Basement” and “No Basement” variables are only valid for detached and non-detached houses. Features representing the micro-location and macro-location are latitude and longitude, different regiotypes, micro score and macro score of a location.

The regiotype was provided by Acxiom, and clusters Germany into ten different area types. In general, Acxiom defines four different spatial types: “Central-Business-District”, “Agglomeration Area”, “Urban Area” and “Rural Area”. The last three can be divided further into three sub-categories each (“Upper Centers”, “Middle Centers”, “Commuter Belt”). All addresses in Germany can be allocated to one of the ten area types. The individual area types are determined according to the respective settlement structure and population density within the municipality and its surrounding area. In most cases, the selected NUTS‑3 regions can be divided further into different Regiotypes and therefore, the integration of different subtypes enables taking further local fixed effects into account.

The micro score of a location is calculated via a gravity model and reflects accessibility in the sense of proximity to selected everyday destinations. A gravity model is a common method for approximating the accessibility of a location and is based on the assumption that nearby destinations play a greater role in everyday life than more distant ones (Handy and Clifton 2001). The score is mainly used to reduce dimensionality and complexity for the EXF. The relevant points-of-interest (POIs) are selected from the findings of Powe et al. (1995), Metzner and Kindt (2018), Yang et al. (2018), Nobis and Kuhnimhof (2018) and Huang and Dall’erba (2021) and are provided in Table 3. A more detailed description of the construction of the micro score of a location can be found in Appendix I.

**Table 3:** Features of the micro score of a location

Points-of-Interests	Category	Description
University	Education & Work	University campus: institute of higher education
School	Education & Work	Facility for education
Kindergarten	Education & Work	Facility for early childhood care
CBD	Education & Work	Center of the next city
Supermarket	Local Supply	Supermarket—a large store with groceries
Marketplace	Local Supply	A marketplace where goods are traded daily or weekly
Chemist	Local Supply	Shop focused on selling articles for personal hygiene, cosmetics, and household cleaning products
Bakery	Local Supply	Place for fresh bakery items
ATM	Local Supply	ATM or cash point
Hospital	Local Supply	Facility providing in-patient medical treatment
Doctors	Local Supply	Doctor’s practice/surgery
Pharmacy	Local Supply	Shop where a pharmacist sells medications
Restaurant	Leisure & Food	Facility to go out to eat
Café	Leisure & Food	Place that offers casual meals and beverages
Park	Leisure & Food	A park, usually urban (municipal)
Fitness Centre	Leisure & Food	Fitness centre, health club or gym
Movie Theater	Leisure & Food	Place where films are shown
Theater	Leisure & Food	Theatre where live performances take place
Shopping Mall	Leisure & Food	Shopping centre—multiple stores under one roof
Department Store	Leisure & Food	Single large store selling a large variety of goods
Subway Station	Transportation	City passenger rail service
Tram Station	Transportation	City passenger rail service
Railway Station	Transportation	Railway passenger only station
Bus Stop	Transportation	Bus stops of local bus lines
E‑Charging Station	Transportation	Charging facility for electric vehicles

*Note:* The descriptions of the selected Points-of-Interest is based on the explanations of Open Street Map. (See https://wiki.openstreetmap.org/wiki/Map_features.)

To account for further local fixed effects, a macro score of a location is computed. For calculation, we use a social area analysis introduced by Carpenter et al. (1955). The method assumes that a city or region can be divided into homogeneous sub-areas on the basis of different environmental variables. The variables used in our study can be seen in Table 4 and are available at ZIP code level. The feature selection is based on Metzner and Kindt (2018). Further information about the macro scores can be found in Appendix II.

**Table 4:** Features for the macro score of a location

Feature	Category	Description
Educational Level	Social Status	Household structure by educational qualifications
Unemployment Rate	Social Status	Proportion of unemployed
Proportion of Children	Social Status	Proportion of population under 6 years
Purchasing Power	Economic Status	Purchasing power per household
Income Structure	Economic Status	Household structure by income
Social Security	Economic Status	Proportion of employees with social security
Relocation Behavior	Real Estate Market	Difference between inflows and outflows
Population Forecast	Real Estate Market	Population forecast for the next 5 years
Building Permits	Real Estate Market	Proportion of building permits
Construction Completions	Real Estate Market	Proportion of construction completed
Time-on-Market	Real Estate Market	Time-on-Market of properties sold

## Methodology

### Expert function

The EXF uses different filters and similarity functions to determine nearby and similar comparable properties. As a result, it provides a final list of *m* comparables, revealing the highest degree of similarity to the property being evaluated. The next step is to estimate the market value by taking the average value of these comparables. Overall, this approach replicates the practice of traditional real estate appraisers in an automated manner. Starting with a total of *N* observations, a filter for spatial proximity is applied first for the EXF. Only observations within a radius of 20 km from the property to be valued are considered. Second, objects are only selected if they have the same Acxiom regiotype. Third, another filter is used to eliminate observations whose valuation date is too far in the past (< 5 years).^5^ Other filters are set for the object type, occupation and presence of a basement, so as to select only corresponding observations. Finally, filters are set for condition and quality grade, eliminating any observations that deviate by more than one category.

After the filtering, $n\leq \mathrm{N}$ observations are left and compared with the object to be valued $x^{*}$ with the aid of similarity functions. These are intended to reflect the appraiser’s approach to the selection of similar properties and make it possible to select only the most similar observations for the final estimation of market value.

First, a function for spatial proximity $SP(x_{i}{,}x^{*})$ is applied for all objects *x*_*i*_, $i\in n$:

$$SP\left(x_{i}{,}x^{*}\right)=\begin{cases} 100-5\cdot d\left(x_{i}{,}x^{*}\right){,}\mathrm{if}\;d\left(x_{i}{,}x^{*}\right)\in \left[0;20\right]{,}\\ 0{,}\text{else}{,} \end{cases}$$

where $d\left(x_{i}{,}x^{*}\right)$ measures the distance between the objects as a network distance measure in kilometers (km). Next, a triangular function for measuring the similarity of the remaining features is applied:

$$tr\left(x_{i{,}f}{,}{x}_{f}^{*}{,}a\right)=\begin{cases} 100-a\left(\left| x_{i{,}f}-{x}_{f}^{*}\right| \right){,}\mathrm{if}\left| x_{i{,}f}-{x}_{f}^{*}\right| < \frac{100}{a}{,}\\ \ 0{,}\text{else}{,} \end{cases}$$

with *x*_*i*,*f*_ being the value of feature *f* of observation *i* and ${x}_{f}^{*}$, the corresponding features of the object being evaluated. *a* describes the slope of the function. A set of different slopes was tested to find the best parameters, yielding *a* to be 10 for the following features: construction year, modernization year, micro score and macro score and 25 for living area and plot size.

For all objects *n*, we are now able to compute the feature-related similarities. These are used to calculate the overall similarity score between all *x*_*i*_ and $x^{*}$:

$$s\left(x_{i}{,}x^{*}\right)=SP\left(x_{i}{,}x^{*}\right)\cdot w_{1}+{\sum }_{f=2}^{7}tr\left(x_{i{,}f}{,}{x}_{f}^{*}{,}a\right)\cdot w_{f}{,}\quad i\in \left\{1{,}\ldots {,}n\right\}{,}$$

with $w_{1}=\frac{1}{7}$ and $w_{f}=\frac{1}{7}$, for all $f\in \left\{2{,}\ldots {,}7\right\}.$

Now, we have the similarity score of the finally filtered objects *n*. The next step is to find the *m* most similar objects to $x^{*}{,}m\leq n.$ Therefore, we construct a new vector *v*, that includes the objects in a sorted manner, so that the object with the highest overall similarity score is in the first entry and the object with the lowest overall similarity score is in the last entry. Only the first *m* objects of *v*, and therefore *m* most similar objects, are considered to evaluate the estimated market value of $x^{*}$ by averaging their market values:

$$f\left(x^{*}\right)=\frac{1}{m}{\sum }_{i=1}^{m}f\left(x_{i}\right).$$

In this paper, the five most similar objects are used to estimate the market value of $x^{*}$, which is the minimum number of comparables required by law to perform a valuation by the sales comparison approach in Germany.^6^

### Ordinary least square regression—OLS

The first hedonic method we use is an OLS. This approach is the most commonly applied hedonic model and often used as a benchmark. Due to its simple architecture, it is easy to understand and interpret. The aim of an OLS is to explain a dependent variable *y*_*i*_ with independent variables $x_{i{,}1}{,}\ldots {,}x_{i{,}k}$ and an error term *ε*_*i*_:

$$y_{i}=\beta _{0}+\beta _{1}x_{i{,}1}+\ldots +\beta _{k}x_{i{,}k}+\varepsilon _{i}{,}$$

for all observations $i=1{,}\ldots {,}n{,}$ with

$$\mu _{i}=E\left[y_{i}\right]=\beta _{0}+\beta _{1}x_{i{,}1}+\ldots +\beta _{k}x_{i{,}k}.$$

Thereby, the unknown parameters $\beta _{1}{,}\ldots {,}\beta _{k}$ are estimated. The OLS assumes that the relationship between the dependent variable and independent variables is linear in parameters. Furthermore, the error terms *ε*_*i*_ are considered to be independent and to have a constant variance. A more detailed description can be found in Fahrmeir et al. (2013).

In order to compare the performance of the models in due course, various optimizations of the OLS are carried out. To achieve the best possible prediction power, several statistical instruments like variable transformations, interaction terms and backward stepwise regression are applied. In contrast to modern machine learning models, these optimizations must be performed manually. With 36 independent variables in the model, 630 pairwise interactions result, which must be calculated and considered for 327 different districts, summing to roughly 206,010 interactions overall. This number can easily go into the millions when higher order interactions are also taken into account. This can be seen as a drawback of the OLS models.

### Generalized additive model—GAM

The GAM is a further development of the OLS and mainly based on the concept behind the Generalized Linear Model. The relationship between the expected value of the dependent variable and the independent variables can be modelled using a monotonic link function *g*, like the logarithm or the identity function. In addition, the GAM has the advantage of being able to include unspecified, non-parametric smoothing functions *s*_*j*_, $j\in \{1{,}\ldots {,}k\}$, of covariates. Consequently, we obtain the model:

$$g\left(\mu _{i}\right)=\beta _{o}+s_{1}\left(x_{i{,}1}\right)+\ldots +s_{k}\left(x_{i{,}k}\right).$$

The main advantage of the GAM compared to the OLS is its flexibility to model non-linear relationships. For the interested reader, we refer to Wood (2017).

Again, to account for locational differences, a combination of different statistical instruments like interaction terms and this time, additionally, different penalized spline types like cubic and thin plane splines have been used. Like the OLS, however, the GAM has the disadvantage that optimizations, such as the choice of spline function or interaction terms, must mainly be performed manually.

### Extreme gradient boosting—XGBoost

Extreme Gradient Boosting is a tree-based ensemble learning method. The idea of ensemble learning algorithms is to combine many so-called weak learners *h*_*m*_, in our case, single decision trees, into one strong learner *h*:

$$h\left(\boldsymbol{y}| \boldsymbol{x}\right)={\sum }_{i=1}^{M}u_{m}h_{m}\left(\boldsymbol{y|x}\right){,}$$

where *u*_*m*_ is used to weight the weak learners. *M* is the number of single trees, x is the full features space and y the response variable. Boosting is a type of ensemble learning in which the weak learners *h*_*m*_ are trained sequentially. Starting with one tree, the subsequent models learn from the previous errors. Gradient boosting uses the so-called gradient descent algorithm by adding new trees to minimize the loss of the model. The eXtreme Gradient Boosting is a computationally effective and highly efficient version of Gradient Boosting. In comparison to parametric and semi-parametric models, the XGBoost detects automatically complex patterns like non-linearities or higher-order interaction terms within a large amount of data, requiring for less manual optimization to account for location differences compared to the OLS and GAM. For more information about tree-based methods, ensemble learning and gradient boosting, the interested reader is recommended to read Hastie et al. (2001).

### Testing concept

**Fig. 2:**
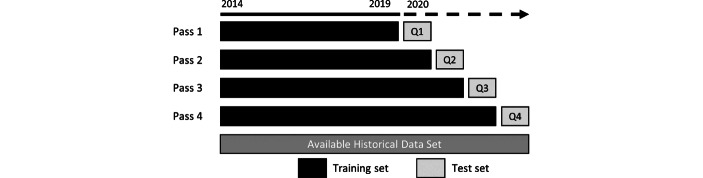
Extending window approach

To evaluate the predictive performance of the models, an extending window approach is implemented according to Mayer et al. (2019). Fig. 2 illustrates the testing concept.

The first iteration divides the dataset into a training set with observations from Q1/2014 to Q4/2019 and a test set from Q1/2020. In the next steps, the newly available data is added to the training set, and the models are retrained and tested on data of the next quarter. The advantages of this approach are that all algorithms are tested on unseen data and thus produce unbiased, robust results. Furthermore, the testing approach provides a realistic testing scenario. In Table 5, the number of training and test observations for each iteration are presented.

**Table 5:** Training and test observations

Data split	Q1	Q2	Q3	Q4
Training	1,063,426	1,106,866	1,141,612	1,180,741
Test	43,440	34,746	39,129	31,805

### Evaluation metrics

For each model, we compute the Mean Absolute Percentage Error (MAPE) and the Median Absolute Percentage Error (MdAPE) as accuracy measures. Unlike Mayer et al. (2019), we use the relative rather than the absolute measures of error to enable a more accurate comparison between administrative districts. Compared to the absolute measures, the relative measures provide a statement that represents the economic loss caused by the application of the algorithms much more precisely, which is very useful in our case, as we conduct a nationwide analysis involving many areas with varying levels of property market values. As Rossini and Kershaw (2008) and Ecker et al. (2020) state, the MAPE and MdAPE are two precision metrics, which enable a useful comparison across different models, datasets and locations. Other examples of their use can be found, for example, at Peterson and Flanagan (2009), Zurada et al. (2011), McCluskey et al. (2013) and Schulz et al. (2014) and Oust et al. (2020).

At this point it should be mentioned that the economic loss for mortgage lenders is not symmetric as overvaluations in particular play a more critical role than undervaluations. Overvaluations significantly increase the potential risk that the value of a property does not cover a mortgage default (see e.g., Shiller and Weiss (1999)). Both the MAPE and the MdAPE are not able to detect if there is a bias in a certain direction. To cover this topic, we additionally analyze a density plot of the relative deviations of the market values to the predicted values to investigate whether there is a bias in a certain direction or not.

In order to obtain an overall picture of the strengths and weaknesses of the algorithms, we additionally provide the proportion of predictions within 10 and 20% (PE(x)), as well as the coefficient of determination R^2^. The ratio of error buckets (PE(x)) allows us to interpret the results in a simple and intuitive way for the human brain. They show how many of the observations can be estimated within a relative deviation of 10 or 20%. Schulz and Wersing (2021) state that the error buckets are frequently used by practitioners when assessing valuation accuracy. A detailed description of all metrics can be found in Table 6.

**Table 6:** Evaluation metrics

Error	Formula	Description
Mean Absolute Percentage Error (MAPE)	$MAPE\left(y{,}\hat{y}\right)=\frac{1}{n}{\sum }_{i=1}^{n}\left| \frac{y_{i}-\hat{y}_{i}}{y_{i}}\right|$	Mean of all absolute percentage errors. A lower MAPE signals higher prediction accuracy in percent
Median Absolute Percentage Error (MdAPE)	$\textit{MdAPE}\left(y{,}\hat{y}\right)=\textit{median}\left({\sum }_{i=1}^{n}\left| \frac{y_{i}-\hat{y}_{i}}{y_{i}}\right| \right)$	Median of all absolute percentage errors. A lower MdAPE denotes a higher precision in percent without being sensitive to outliers
Error buckets (PE(x))	$PE\left(x\right)=100\left| \frac{y_{i}-\hat{y}_{i}}{y_{i}}\right| < x$	Percentage of predictions where the relative deviation is less than *x%*, with *x* being 10 and 20. A larger PE(x) signals a lower variation in the predictions
R^2^	$R^{2}\left(y{,}\hat{y}\right)=1-\frac{{\sum }_{i=1}^{n}\left(y_{i}-\hat{y}_{i}\right)^{2}}{{\sum }_{i=1}^{n}\left(y_{i}-\overline{y}\right)^{2}}$	Coefficient of determination. A high R^2^ is an indication of better goodness of fit of the model

## Results

### Results at national level for Germany

Firstly, the models are compared at a national level. In Table 7, the prediction errors of the entire year 2020 are summarized. For all methods, the results of the metrics evolve similarly. The more complex the structure of the approach, the better the performance. The EXF is designed to replicate the practice of traditional real estate appraisers in an automated manner and is therefore readily understandable. However, the approach provides the poorest results. Comparing these results with the performance of the OLS, often used as a baseline model, we can see a performance improvement. Relatively speaking, the MAPE of the OLS is around 18% lower and the MdAPE 19%. In addition, using an OLS results in 18% and 20% more predictions deviating less than 10 and 20% from their actual market value.

**Table 7:** Model prediction errors 2020 throughout Germany

Models	MAPE	MdAPE	PE(10)	PE(20)	R^2^
EXF	0.2130	0.1624	0.3267	0.5872	0.7735
OLS	0.1736	0.1311	0.3937	0.6940	0.8654
GAM	0.1646	0.1202	0.4273	0.7276	0.8664
XGB	0.1465	0.1084	0.4665	0.7786	0.8995

Analyzing the results of the GAM, we again see a boost in performance compared to the OLS. But this time the relative improvement is smaller. The MdAPE of the OLS is around 9% higher. In addition, the percentage of predictions with a relative deviation of less than 10 and 20% increased by 9% and 5% respectively. This might be caused by the ability of the GAM to model more complex non-linearities within the data, which is extremely difficult to manually reproduce within the OLS, and practically impossible to implement for 327 districts. This is especially so, since these manual adaptions have to be done in each of the four quarters.

Overall, the XGBoost yields the best model performance regarding all evaluation metrics due to its ability to capture and process joint effects, non-linear relationships and high-dimensional structures within the data with comparably low manual effort. Comparing the results of the XGBoost with the EXF 43% and 33%, more observation deviate less than 10 and 20% from their market values.

**Fig. 3:**
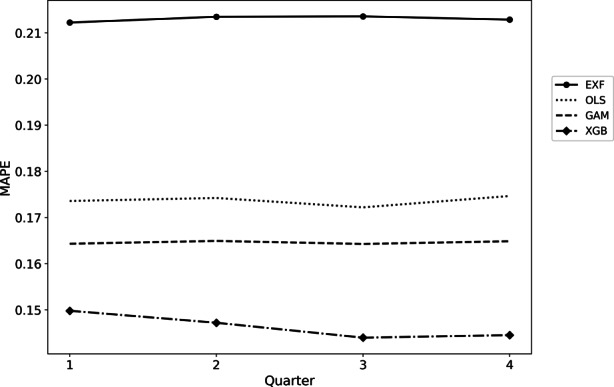
Quaterly model error performance based on MAPE

The chosen extending-window testing approach allows us to further analyze the performance of all four algorithms over the four quarters of 2020. Confirming the previous results, the solid line in Fig. 3 shows the trends already mentioned. Additionally, it is interesting how consistently the models perform over all four quarters. Moreover, the XGBoost displays better performance the more training data it can process. The exact numbers can be seen in Appendix III.

One research question of this study is to determine whether modern machine learning methods are able to outperform traditional hedonic models and the EXF approach. Analyzing our results at the national level for Germany we can clearly confirm this. The XGBoost yields a significant performance improvement compared to the EXF, OLS and GAM. This shows that in the future, regulators should also discuss the approval of machine learning methods in the field of AVMs. The application of machine learning approaches can lead to a reduction in the economic loss caused by the AVM. Machine learning algorithms are able to better assess possible risks within the lending process and can thus fulfill the actual purpose of a real estate valuation in a much more target-oriented manner.

### Results at the administrative district level

**Fig. 4:**
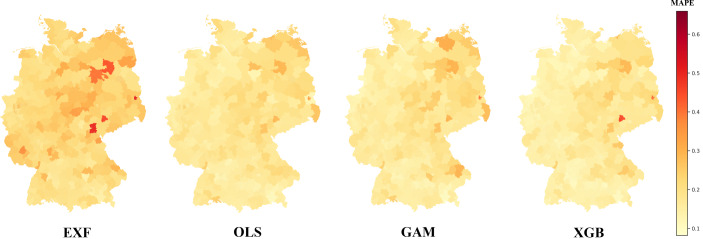
Error comparison at administrative district level

After comparing the models at the national level, we want to examine the model performance in more detail. Therefore, we focus on the level of the 327 administrative districts. In Fig. 4, the performance based on the MAPE for the different methods is shown cartographically. The maps confirm the abovementioned trends. The EXF again yields the overall poorest performance and again, it can be seen that the more complex the approach, the better the results. In addition, all four models are unsatisfactory with respect to estimating the market value in the same administrative districts. This can also be confirmed by the correlation matrices shown in Appendix III. Especially in the eastern part of Germany, the MAPE tends to be higher. This result might be caused by the lower data availability in these regions.

**Fig. 5:**
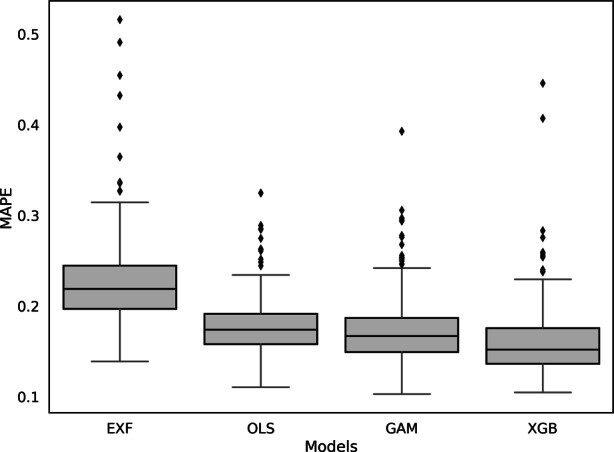
Box plots of MAPE at administrative district level

To obtain a better understanding of the model performance at the administrative district level, we focus on the box plots of the MAPE in Fig. 5. Those confirm the trend displayed in Fig. 4. The EXF again yields the overall poorest results. It delivers the largest interquartile range, the longest whiskers and contains the most outliers. The XGBoost has the lowest median MAPE of all four models, whereas it has only two extreme outliers. In contrast, the GAM and especially the OLS have a smaller range of outliers. These results indicate that the XGBoost does not always display the best model performance and therefore, different models should be used for each administrative district.

Table 8 shows the percentage of the administrative districts for which each model performs best. The XGBoost yields the best performance in all metrics for most administrative districts. Focusing on the hedonic approaches, the GAM and OLS are also superior in some regions, whereas EXF is the least convincing. The analysis shows that, in the case of Germany, there is no universally valid model that performs best in all administrative districts. Instead, it is advisable to apply different models in different regions.

**Table 8:** Model performance at administrative level

Models	MAPE	MdAPE	PE(10)	PE(20)	R^2^
XGB	0.7920	0.7187	0.6636	0.6636	0.6422
GAM	0.1162	0.1988	0.2202	0.2202	0.0550
OLS	0.0826	0.0765	0.1101	0.1101	0.2997
EXF	0.0092	0.0061	0.0061	0.0061	0.0031

**Fig. 6:**
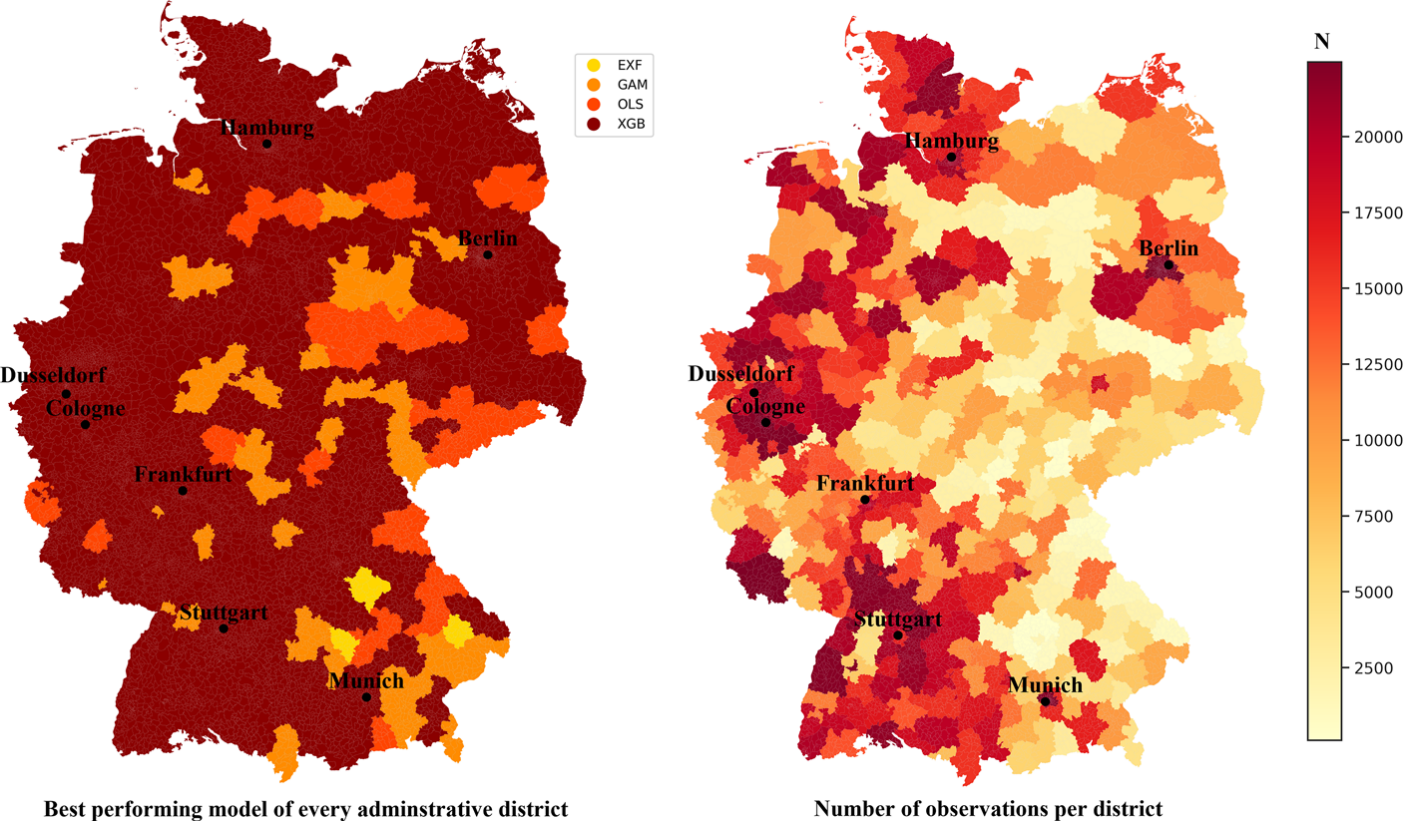
Model performance and number of observations per administrative district

To gain a deeper understanding of the finding that different models should be used in different regions, it is useful to present the results cartographically. On the left side of Fig. 6, the best performing model regarding the MAPE in the administrative districts is shown. On the right, the number of observations per district is presented.

In the north, west and south-west of Germany, the XGBoost shows the best model performance. In contrast, especially in the south-east and east, a different picture emerges. Comparing the availability of observations with these findings, a clear dependence can be derived. In areas with many observations, the XGBoost in particular can demonstrate its strengths. By contrast, in areas with only a few observations—mostly rural regions—the GAM and OLS can also convince. Consequently, especially if one aims to implement an AVM including several different locations with a different amount of data, multiple algorithms have to be considered. By testing different algorithms, the specifics of each region can be addressed, and thus, the best model for each region can be used. This ultimately leads to a reduction of the economic loss caused by the AVM. This result shows that regulators should generally consider approving of different algorithms, and that their focus should not be on only one type of procedure.

### Results at the prediction level

**Fig. 7:**
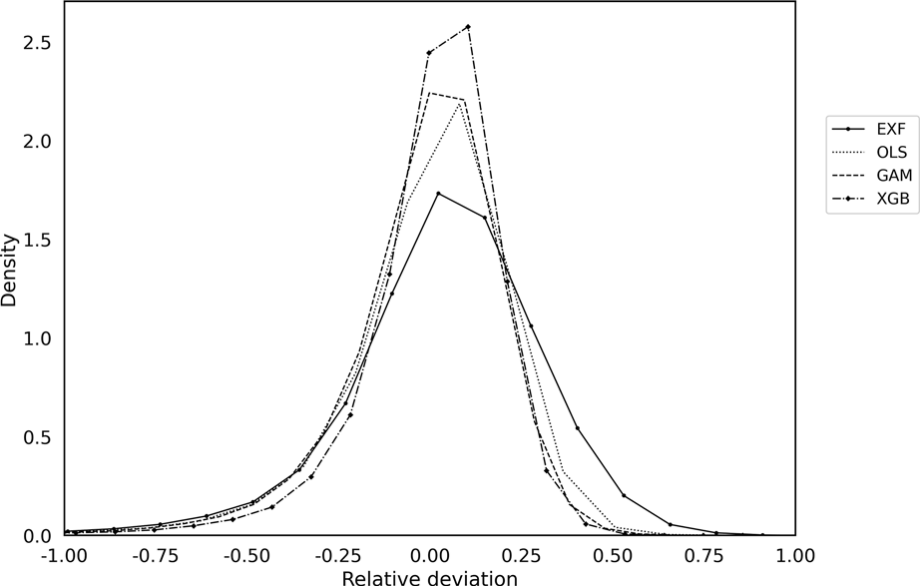
Density plot of the relative deviation of the market values to the predicted values

Lastly, we analyze the relative deviations of the market values to the predicted values for all four models. In addition to the known evaluation metrics, with regard to the regulatory requirements, it is recommended to always perform an analysis at the prediction level to check whether overvaluations and undervaluations occur evenly, or whether the algorithms used exhibit a bias in one direction. In terms of choosing the right model from a practitioner’s perspective, this can have a big impact and reduce financial risks from automated valuations in the long run. Accordingly, Fig. 7 provides the density plots at the prediction level. It is evident that the EXF is negatively skewed, indicating that the approach underestimates market values to a greater extent. Transferring this point to practice shows that the use of the EXF may be more advantageous from a risk management perspective, since the economic loss caused by an incorrect estimate by the model is statistically lower. In the event of a loan default and a potential undervaluation by the EXF, the outstanding loan amount should more easily be recovered from the proceeds of a foreclosure sale than it would be the case if the property were overvalued. The curves of the OLS, GAM and the XGBoost are more symmetric and rather leptokurtic. This suggests that overvaluations and undervaluations occur more evenly, potentially increasing the risk of economic loss relative to the EXF.

**Fig. 8:**
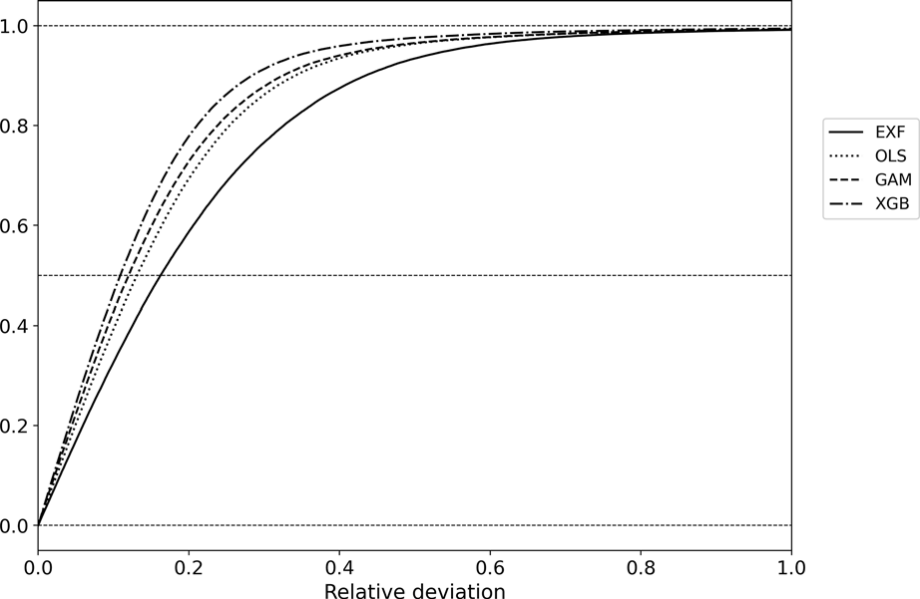
Cumulative distribution function plot of the relative deviation of the market values to the predicted values

Furthermore, a cumulative distribution function plot, shown in Fig. 8, is used to reveal whether one method outperforms another stochastically. The XGBoost is superior to the other models, with the GAM and OLS in particular being very close. In contrast, a clear gap can be seen between the OLS and the EXF. This confirms the results from above, and shows again that it is important from the regulator side also to think about approving of machine learning methods in the area of AVMs.

## Conclusion

This study compares different approaches to constructing AVMs on a nation-wide level in order to provide empirical evidence on the regulatory debate on the future use of automated valuations. In particular, we answer the question of whether more thought should also be given to the future use of machine learning algorithms in the context of AVMs. For this purpose, an automation of the sales comparison method by using filters and similarity functions—the EXF, two hedonic price functions based on OLS and GAM, as well as the machine learning approach XGBoost, are implemented for 327 administrative districts in Germany.

As our results show, the machine learning approach XGBoost achieves the highest overall accuracy (MAPE, MdAPE, PE(10), PE(20), R^2^) in the valuation of standard residential properties in Germany. One reason might be its ability to automatically capture and process joint effects, non-linear relationships and high-dimensional structures within a large number of observations, without requiring as many manual optimizations to account for location differences. Therefore, the XGBoost convinces in practice with its flexibility. Especially in the metropolitan areas with many observations, the relationships between the variables determining the market value seem to be much more complex, implying a need for more complex valuation models. The OLS and GAM yield weaker results. Several optimizations have been carried out to increase their predictive performance and to ensure the comparability of the models as well. However, practical application shows that the optimization of the well-established methods is time-consuming, labor-intensive and in particular, therefore shows significant disadvantages in the implementation for 327 individual districts, as it is practically infeasible. Also, the EXF does not come close to the performance of the XGBoost. The EXF even shows the weakest performance compared to the XGBoost, the OLS and the GAM. Our results indicate that the EXF tends on average to underestimate the predicted market values.

Furthermore, the results of our study show that for designing an AVM, there is no “one size fits all”. Although the XGBoost is the best performer across the country, there are also administrative districts where the EXF, OLS, or GAM are best suited for estimating market values. In this context, it is particularly evident that the respective data availability seems to play a role. In districts with fewer observations, the traditional approaches manage to outperform the modern machine learning approach. In order to take this into account and to optimize the overall performance of AVMs, regulators should not merely allow, but actively promote the use of different types of algorithms. Before finally deploying an AVM, different types of methods should be tested for each district.

In the field of lending, a mispricing has major implications for both lenders and borrowers. Accurate model estimates are of considerable importance to ensure the resilience of the banking sector, especially in crisis periods. Our results clearly show that the approval of machine learning algorithms should be considered by regulators. We believe that machine learning algorithms have a high degree of robustness and resilience and are therefore ideally suited for AVMs. The traceability and auditability of the results required by the supervisory authorities can also be ensured by using the latest methods from the field of eXplainable Artificial Intelligence (XAI). While machine learning algorithms were considered as black box for a long time, XAI methods, like SHapely Additive exPlanations (SHAP) plots or Accumulated Local Effects (ALE) plots, are able to decode the basic decision-making process of any machine learning model. XAI is still at an early stage in the field of real estate research, but we are convinced that this will change in the coming years, and that new and important insights will be generated, which will further confirm the advantages of the use of machine learning algorithms. We therefore recommend re-examining the debate on the use of AVMs in everyday appraisals and, in particular, also including new and innovative methods.

## Figures and Tables

**Fig. 9:**
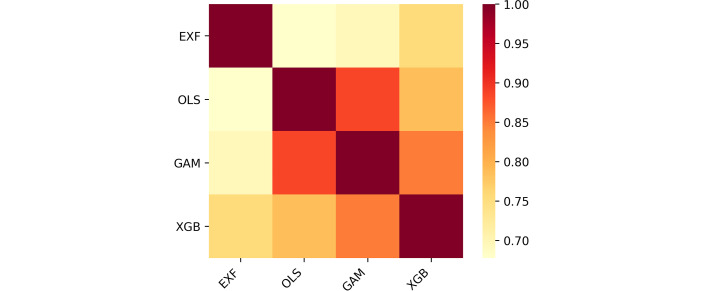
District error correlation across the models

## Appendix

### Appendix I—Micro score

Our gravity model can be described using an activity function *f*(*A*_*p*_) and a distance function *f*(*D*_*i*,*p*_):

$$A_{i{,}p}=\sum f\left(A_{p}\right)f\left(D_{i{,}p}\right).$$

$A_{i{,}p}\in \left[0{,}100\right]$ denotes the accessibility of point *i* for the POI *p*, whereby the activity function *f*(*A*_*p*_) specifies the relative importance of POI *p*, with *f*(*A*_*p*_ ∈ [0,1]. *f*(*D*_*i*,*p*_) measuring the travel time from point *i* to the POI *p* by using a non-symmetric sigmoidal distance function. The travel time was obtained for the selected POIs via Open Street Map and normalized using the following function:

$$L\left(x\right)=\frac{K}{\left(1+Qe^{0.5x}\right)^{\frac{1}{v}}}{,}$$

where $K{,}Q\in \mathbb{R}$ and $v\in R^{+}$ are defined for all possible distances $x\in \mathbb{R}.$ Furthermore, we have:

$$K=\left(1+Q\right)^{1+v}{,}$$

$$Q=v\cdot \exp \left(B\cdot x^{*}\right){,}$$

$$v=\frac{\exp \left(B\cdot x^{*}\right)-1}{\ln \left(y_{i}\right)-1}{,}$$

where $x^{*}$ denotes a feature specific point of inflection and $y^{*}$ is 0.5.

### Appendix II—Macro score

The scores *V*_*j*,*i*_(*z*) for each variable *z* in ZIP code *i* of region *j* are calculated using the following function:

$$V_{i{,}j}\left(z\right)=\left(\frac{100}{\max (z_{j})-\min (z_{j})}\right)\left(z_{i}-\min \left(z_{j}\right)\right){,}$$

where *z*_*i*_ denotes the value of feature *z* of ZIP code *i*. max(*z*_*j*_), and min(*z*_*j*_) are the maximum and minimum values of feature *z* in region *j*. As *j*, we define the 327 available administrative districts. Individual scores for all variables *z* included in the macro scores are calculated. The final macro score *MAS*_*i*,*j*_ is computed by averaging the single scores in ZIP code *i*:

$$MAS_{i{,}j}=\frac{1}{\left| z\right| }\sum _{z}V_{i{,}j}\left(z\right).$$

### Appendix III—MAPE results on a quarterly basis

**Table 9:** MAPE on a quarterly basis throughout Germany

Models	Q1	Q2	Q3	Q4
EXF	0.2122	0.2135	0.2136	0.2129
OLS	0.1736	0.1742	0.1722	0.1747
GAM	0.1643	0.1649	0.1643	0.1649
XGB	0.1498	0.1472	0.1440	0.1445

## Footnotes

1 Acxiom is an American provider of international macroeconomic and microeconomic data. Further information can be found at: https://www.acxiom.com/.

2 Berlin, Munich, Hamburg, Frankfurt am Main, Cologne, Dusseldorf, Stuttgart.

3 The correlation matrix is available on request.

4 Applies if the property is both partly owner-occupied and partly non-owner-occupied (e.g., single-family home with attached rental unit).

5 For valuations longer than one year ago, an indexation with the Destatis Real Estate Price Index is applied. The index is available quarterly for five Destatis-Regiotypes starting in 2016. Mapping with the Acxiom Regiotype is performed. Further information about the index can be found at https://www-genesis.destatis.de/genesis/online.

6 This procedure is based on the German guidelines for determining the mortgage lending value, see §4 BelWert.
